# Analysis of Major Genome Loci Underlying Artemisinin Resistance and *pfmdr*1 Copy Number in pre- and post-ACTs in Western Kenya

**DOI:** 10.1038/srep08308

**Published:** 2015-02-06

**Authors:** Bidii S. Ngalah, Luiser A. Ingasia, Agnes C. Cheruiyot, Lorna J. Chebon, Dennis W. Juma, Peninah Muiruri, Irene Onyango, Jack Ogony, Redemptah A. Yeda, Jelagat Cheruiyot, Emmanuel Mbuba, Grace Mwangoka, Angela O. Achieng, Zipporah Ng'ang'a, Ben Andagalu, Hoseah M. Akala, Edwin Kamau

**Affiliations:** 1 Department of Emerging Infectious Diseases-Global Emerging Infections Surveillance and Response System (DEID-GEIS) Program, United States Army Medical Research Unit-Kenya (USAMRU-K), Kenya Medical Research Institute (KEMRI)-Walter Reed Project, Kisumu, Kenya; 2 Institute of Tropical Medicine and Infectious Diseases, College of Health Sciences, Jomo Kenyatta University of Agriculture and Technology, Nairobi, Kenya; 3 School of Biological/physical sciences, Department of Zoology, Maseno University, Maseno, Kenya; 4 Ifakara Health Institute, Bagamoyo, Tanzania

## Abstract

Genetic analysis of molecular markers is critical in tracking the emergence and/or spread of artemisinin resistant parasites. Clinical isolates collected in western Kenya pre- and post- introduction of artemisinin combination therapies (ACTs) were genotyped at SNP positions in regions of strong selection signatures on chromosome 13 and 14, as described in Southeast Asia (SEA). Twenty five SNPs were genotyped using Sequenom MassArray and *pfmdr*1 gene copy number by real-time PCR. Parasite clearance half-life and in vitro drug sensitivity testing were performed using standard methods. One hundred twenty nine isolates were successfully analyzed. Fifteen SNPs were present in pre-ACTs isolates and six in post-ACTs. None of the SNPs showed association with parasite clearance half-life. Post-ACTs parasites had significantly higher *pfmdr*1 copy number compared to pre-ACTs. Seven of eight parasites with multiple *pfmdr*1 were post-ACTs. When in vitro IC_50_s were compared for parasites with single vs. multiple gene copies, only amodiaquine and piperaquine reached statistical significance. Data showed SNPs on chromosome 13 and 14 had different frequency and trend in western Kenya parasites compared SEA. Increase in *pfmdr*1 gene copy is consistent with recent studies in African parasites. Data suggests genetic signature of artemisinin resistance in Africa might be different from SEA.

The emergence and/or spread of drug resistant *Plasmodium falciparum* parasites continue to be a threat to global malaria control and elimination efforts. This has prompted renewed call to increase and sustain surveillance efforts to monitor emergence and/or spread of resistance^1^. Drug resistance is monitored by conducting in vivo efficacy studies, in vitro and genetic analyses of field clinical isolates. Genetic analysis of molecular markers and in vitro drug susceptibility testing are the preferred methods for surveillance studies because they are practical; a large number of field isolates can be rapidly and inexpensively collected and analyzed compared to in vivo efficacy studies. Genetic analysis of molecular markers has an additional advantage in that blood samples can also be conveniently collected on filter papers as dry blood spots, which can be transported and stored without the need for cold chain for several months^2^.

Molecular markers have been used to make policy decisions^3^ and to monitor changes in parasite drug susceptibility, especially longitudinally after change in treatment policies^4^. However, some of the molecular markers such as those for chloroquine and sulphadoxine-pyrimethamine (SP) resistance did not have much of an impact in predicting the emergence and/or spread of resistance because by the time they were discovered, resistance was already prevalent worldwide. With introduction of artemisinin combination therapies (ACTs) as the first-line treatment in most malaria endemic countries, discovery of molecular markers for resistance before resistance has widely emerged and/or spread will be crucial in surveillance efforts.

Decline in efficacy of ACTs have been reported in Southeast Asia (SEA)^5^,^6^,^7^,^8^. Although the mechanism of resistance and molecular markers to some of the partner drugs such as mefloquine and lumefantrine are already determined^9^,^10^,^11^, the mechanism of reduced susceptibility to artemisinins and the molecular marker that confer resistance has only been recently described^12^. Artemisinin resistance phenotype was attributed to mutations in portions of a *P. falciparum* gene (PF3D7_1343700) encoding K13-propeller domain. However, the mutations in K13-propeller domain that confer resistance in parasites from SEA have not been found in African parasites^13^,^14^,^15^,^16^. This is expected in part because there is no evidence of decline in ACTs efficacy in Africa^16^,^17^. It is also likely that the genetic background and profile of the parasites will influence the molecular marker(s) that are determinant of artemisinin resistance. Even though the parasite population structures and genetic profiles of parasites in SEA is different from those in Africa, progress made in genetic and genome-wide studies in SEA present basis which genetic and genome-wide studies in African parasites can be modeled after^7^,^8^,^12^,^18^,^19^,^20^.

Genome-wide analysis of *P. falciparum* isolates from SEA has shown substantiation of recent positive selection in geographic regions where there is reduced susceptibility to ACTs^8^,^18^. In one of the studies, screening of Single Nucleotide Polymorphisms (SNPs) and microsatellites in samples collected from geographically distinct locations in SEA showed strong selection signatures in regions on chromosome 13 and 14^8^. The selective sweep on chromosome 13 further showed strong association with slow parasite clearance rate (CR) which is considered to be the first evidence of the development of clinically significant resistance. In an attempt to identify genetic basis for adaptive traits and to explore if the same traits seen in SEA might be present and/or relevant in Kenyan parasites, we analyzed SNPs on chromosome 13 and 14 as described by Cheeseman *et al*^8^. Isolates analyzed were collected before (pre-) and after (post-) introduction of artemether-lumefantrine (AL) in Kenya. Further, we analyzed the *P. falciparum* multidrug resistance gene (*pfmdr*1) copy numbers which has been shown to be a key determinant for in vitro reduced sensitivity to several antimalarial drugs as well as treatment failure. We investigated the correlation of *pfmdr*1 to several in vitro drugs and parasite CR.

## Results

### Genetic polymorphism in pre- and post-ACTs parasites

**Figure 1:**
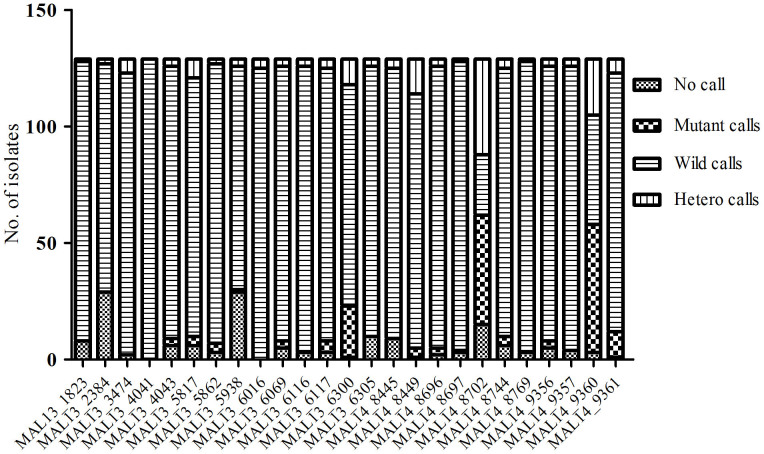
The call rate in each SNP in 129 isolates assayed. 48 samples were pre-ACT samples collected between period (1995–2003), 10 were collected in the post ACT period during transition period (2005–2008) and 71 were from the 2013–2014 in vivo efficacy clinical trial study. The call rates for each SNP (x-axis) in all 129 isolates analyzed is shown.

A total of 129 field isolates were genotyped using the Sequenom MassARRAY in 25 of the 26 SNP positions on chromosome 13 and 14 as described by Cheeseman *et al*^8^. Parasites were successfully genotyped as follows: 48 field isolates collected between 1995-2003 (pre-ACTs), 10 collected during the transition period (2005–2008) and 71 field isolates collected between 2013–2014 (post-ACT). Figure 1 show the genotype call per each assay where the number of successful assays for each SNP is shown. MAL13_4041 SNP assay was the most successful with all 129 samples successfully analyzed whereas MAL13_2384 SNP assay was the least successful with only 89 samples successfully analyzed. MAL14_8702 SNP assay had the largest number of mutant and heterozygous calls (47 mutant calls and 41 hetero calls). SNPs with minor allele frequency (MAF) > 1% were used in the analysis. Table 1 shows the profile of each SNP and the MAF. A total of fifteen SNPs had MAF > 1%. All the fifteen SNPs were present in the pre-ACTs samples whereas only six were present in the post-ACTs samples. The six SNPs present in post-ACTs isolates were all present in the pre-ACT samples. The SNP which occurred with the highest frequency was MAL14_9360, present in 57% of the pre-ACT isolates. The six SNPs that were present in the pre-ACT and post-ACT samples declined in frequency after the introduction of ACTs. MAL10-688956 and MAL13-1718319 as described by Takala-Harrison *et al*^18^ carried only wild type alleles.

### Association of genetic polymorphism with parasite clearance half-life

Sample isolates collected in 2013–2014 were from an in vivo efficacy study where clearance half-life of the parasite infections in a subset of samples in response to AL was obtained. Detailed information of this study including parasite clearance half-life will be reported elsewhere. However, additional information on the study can be found as Supplementary Note online. The mean parasite clearance half-life was 2.63 hours (95% confidence interval [CI], 2.44–2.81) and the median clearance half-life was 2.55 hours (interquartile range [IQR], 1.99–3.21). Proportion of infections with clearance half-life > 2.55 hours was 57%. To investigate the association of genetic polymorphisms in the markers investigated in this study with parasite clearance half-life, the association of the frequency of each SNP to parasite clearance half-life was analyzed. The clearance half-life of 62 parasites isolates was analyzed against the parasite genotype, either as a single SNP or combined. The parasite with the longest clearance half-life of 5.05 hours carried the MAL14_8702 and MAL14_9360 genotypes. Two of the parasites carrying MAL13_6300 and MAL14_8702 genotypes had parasite clearance half-life above 3 hours. However, none of these genotypes single, combined or grouped together (wild type vs. mutant) showed significant association with parasite clearance half-life (p = 0.70 Kruskal-Wallis test).

### Association of genetic polymorphism with in vitro drug susceptibilities

The correlation between parasite genotype(s) and drug IC_50_s for 27 randomly selected post-ACTs samples was evaluated for three artemisinin based compounds and four partner drugs as described in the methods section. Single or various combinations of double genotypes were analyzed using 1-way ANOVA Kruskal-Wallis test against drug IC_50_s whereas wild type vs. all mutant alleles (grouped together) were analyzed using Mann-Whitney test, two-tailed t test against drug IC_50_s. When the association of each SNP (wild type; MAL13_6300; MAL14_8702) or combined SNPs (MAL13_6300 and MAL14_8702; MAL14_8449 and MAL14_9702; MAL14_8702 and MAL149360) with drug IC_50_s were analyzed using the Kruskal-Wallis test, none of the association was significant. Interestingly however, for dihydroartemisinin, the mean (and the median) IC_50_ for the mutant allele(s), either single or combined were 2–3 times as much as those for wild type allele. Further, when Mann-Whitney test was done for wild type vs. all mutant alleles (grouped together), the IC_50_s for artemether and dihydroartemisinin reached statistical significance with p values of 0.027 and 0.008 respectively.

### Distribution of *pfmdr*1 gene copy number in pre- and post-ACTs sample isolates

**Figure 2:**
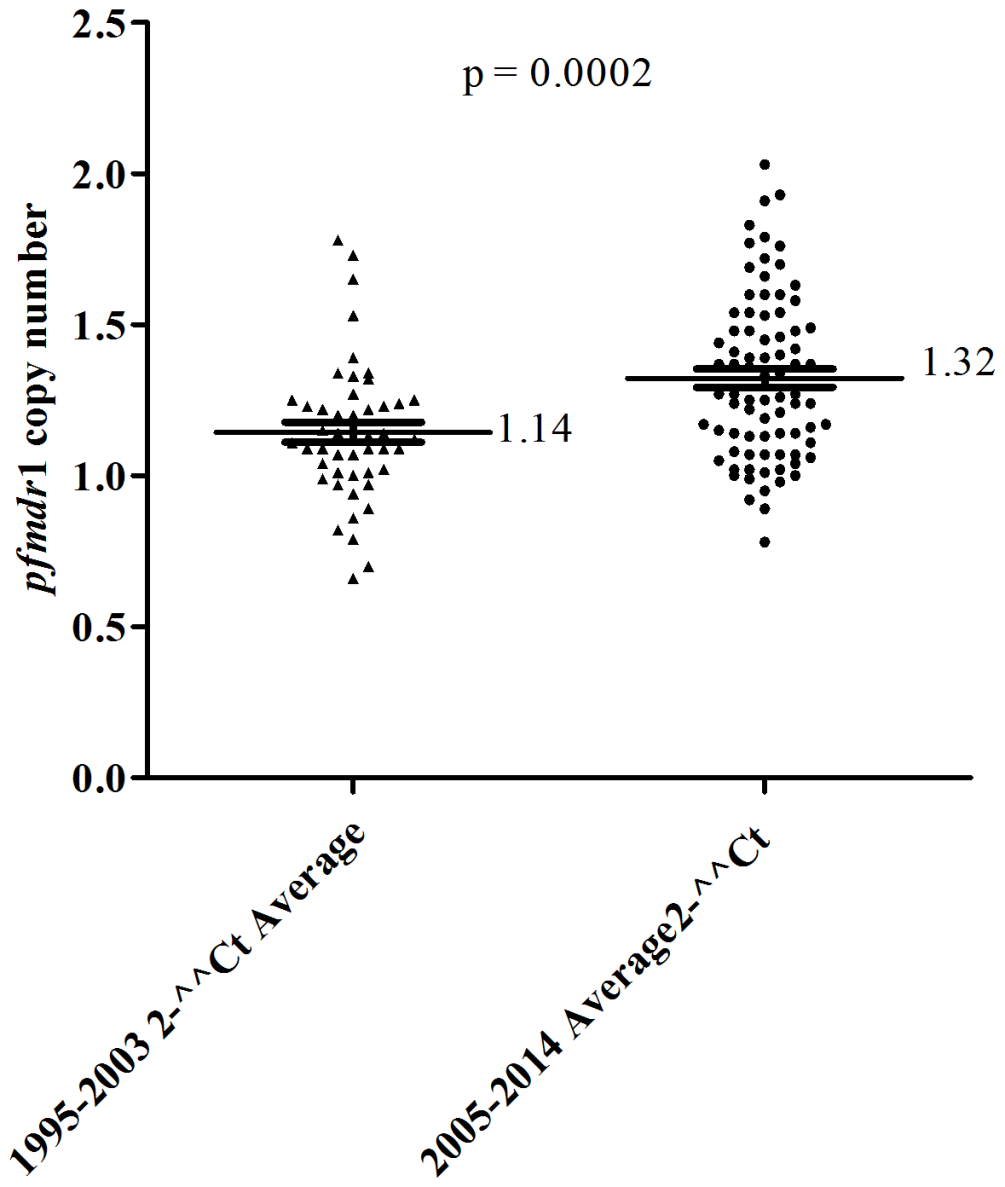
Comparison of the distribution of *pfmdr*1 copy number between pre- and post-ACTs periods. Data showing the mean distribution of 48 samples *pfmdr*1copy number for pre-ACTs samples (1.14) and 71 post-ACT samples (1.32). There was statistical significant difference of *pfmdr*1 gene copy numbers between the two periods based on the t-test (p = 0.0002).

Recent studies have implicated use of ACTs in the increase of *pfmdr*1 gene copy number in African parasites^21^,^22^. To investigate whether there was change in *pfmdr*1 gene copy in Kenyan parasites with the introduction of ACTs, the presence and distribution of copy number variation of the *pfmdr*1 gene in pre- and post-ACTs isolates was evaluated. Of the 129 sample isolates analyzed, eight carried multiple *pfmdr*1 gene copy numbers. Seven of these were post-ACT and one was pre-ACT. The mean *pfmdr*1 gene copy number for the eight samples was 1.85 (95% CI, 1.76–1.93) compared to the single *pfmdr*1 gene copy number which was 1.21 (95% CI, 1.17–1.24). Further, the pre- and post-ACTs *pfmdr*1 gene copy number for all the samples was compared. Figure 2 shows the post-ACTs samples had higher *pfmdr*1 gene copy numbers (p = 0.0002).

### Association of multiple *pfmdr*1 copy numbers with in vitro drug susceptibilities

The correlation between drug IC_50_ and parasite *pfmdr*1 copy number was evaluated for three artemisinin based compounds and four partner drugs. The drug IC_50_s were obtained for 20 randomly selected post-ACTs samples with single *pfmdr*1 copy number and the seven post-ACTs samples with multiple *pfmdr*1 copy number. Table 2 shows the p values obtained after comparing IC_50_s of samples with single and multiple *pfmdr*1 gene copy numbers. There was no significant difference in IC_50_s obtained between single and multiple *pfmdr*1 copy number of any of the artemisinin derivatives. However, for the partner drugs, amodiaquine and piperaquine reached significance with p values of 0.009 and 0.038 respectively. Lumefantrine and mefloquine did not reach significance.

### Association of *pfmdr1* gene copy number with parasite clearance half-life

To investigate whether there was any association between *pfmdr*1 gene copy number and parasite clearance half-life, the clearance half-life of parasite carrying single or multiple copy numbers of *pfmdr*1 gene were compared. The mean clearance half-life of samples with multiple *pfmdr*1gene copy number was slightly elevated at 2.63 hours whereas those with single copy number 2.35 hours; the difference did not reach statistical difference revealing there was no association of *pfmdr*1 gene copy number with parasite clearance half-life.

## Discussion

In this study, we have reported the number and frequency of SNPs in chromosome 13 and 14 of *P. falciparum* genome in samples collected in Kenya during pre- and post-ACTs periods as reported by Cheeseman *et al*^8^. Fifteen SNPs were present in pre-ACTs parasites whereas only six were present in post-ACTs parasites. Interestingly, the frequency of all the SNPs was higher in pre-ACTs parasites than in post-ACTs. There was no association of parasite genotype in any of the SNPs analyzed with the parasite clearance half-life. However, there was significant association between parasite genetic polymorphism with artemether and dihydroartemisinin IC_50_s. We also analyzed resistance-associated alleles at SNPs MAL10-688956 and MAL13-1718319 as described by Takala-Harrison *et al*^18^ but none of the parasites, pre- or post-ACTs carried any of the resistance allele. Data revealed that there was increase in *pfmdr*1 copy number in post-ACTs period compared to pre-ACTs, with seven of eight multiple *pfmdr*1 copy number parasites found in post-ACTs parasites. Further analysis revealed that increased *pfmdr*1 copy number was associated with increased in vitro susceptibility to piperaquine and amodiaquine.

A 35kb locus within chromosome 13 was shown to contain heritable component of slow CR, suggesting it is a major determinant of artemisinin resistance^8^. Some of artemisinin resistant candidate genes within this region of chromosome 13 include lipoate synthase, aminomethyltransferase, hsp70 and other conserved proteins with unknown functions. Some mutations within this region were shown to be present at high frequency in artemisinin resistance parasite population in SEA. However, these mutations were also present in parasites before the emergences of artemisinin resistant parasites suggesting that they might contribute, but do not directly underlie artemisinin resistance. In Kenyan parasite, there was no emergence of new mutation in chromosome 13 with the introduction of ACTs. To the contrary, the number of mutations in chromosome 13 reduced from fifteen to six from pre- to post-ACTs periods. This is partly in line with what was seen in SEA where frequency of mutations was high in both pre- and post-artemisinin resistance parasite populations^8^. The decrease in number and frequency of mutations in chromosome 13 in Kenyan parasites is indicative of release of pressure on the parasite population. Given that there are a large number of candidate genes in this region of chromosome 13 with a wide array of known and unknown cellular functions, it is likely that antimalarial drugs such as chloroquine and SP, the first-line treatment of uncomplicated malaria before the introduction of ACTs in Kenya in 2006, might having been exerting pressure and causing parasite mutations. It is plausible that change of drug policies in Kenya resulted in release of pressure on parasite population which resulted in change (decline) of mutations found in this region of chromosome 13. The existence of these mutations did not offer any benefit to the parasite population which reverted back to the wild type with the release of pressure. Further studies are required to substantiate this observation and determine if mutations in chromosome 13 might be candidate molecular markers for resistance (or pressure) to other drugs, similar to polymorphisms in *pfmdr*1 gene which are molecular markers for resistance to several antimalarial drugs.

Although in vivo efficacy studies are the gold standard method used for tracking drug resistance including clinical artemisinin resistance, these studies require substantial resources. In vitro/ex vivo assays and molecular markers of drug resistance are more affordable, they play a crucial role in tracking resistance^2^, and might allow for the detection of resistance before it is clinically evident. In this study, there was no evidence of significant association between parasite genotype in any of the genetic markers analyzed (single, combined or grouped) with parasite clearance half-life. Interestingly however, there was evidence of some association between genetic polymorphism with in vitro drug susceptibilities. Genetic polymorphisms in *pfmdr*1 have been implicated in altered sensitivity to multiple structurally unrelated antimalarials including artemisinin^9^,^10^,^11^. In addition, polymorphism in other genes including the sarco/endoplasmic reticulum calcium-dependent ATPase (SERCA) homologue *pfATP*6^23^ and multidrug resistance protein homologue, *pfmrp*1^24^ have potentially been associated with artemisinin resistance. Mutations in K13-propeller domain confer resistance in parasites from SEA^12^,^16^. In this study, we have demonstrated some of the SNPs in chromosome 13 and 14 described by Cheeseman *et al*^8^ might potentially be of interest in Kenyan parasites for monitoring resistance to artemisinin compounds. Even though the association of each SNP or combined with IC_50_s did not reach statistical significance for any of the drugs tested, the IC_50_ means (and medians) were high for parasites carrying the mutant genotype(s) compared to the wild type. Further, when grouped, the IC_50_s for artemether and dihydroartemisinin in parasites carrying mutant alleles were significantly higher than those carrying wild type alleles. To verify these findings, it will be important to analyze a large number of samples including single-clone infections and samples from other parts of the country to account for the difference in parasite genetic background. It will be also important to analyze genetic markers described here in the context of other genetic markers that have been implicated in alteration of sensitivity of artemisinin based compounds.

In SEA, increase in *pfmdr*1 gene expression has been associated with reduced sensitivity to several antimalarial drugs including artesunate, mefloquine, artesunate-mefloquine, and artemether-lumefantrine^10^,^11^,^25^. These parasites usually carry 2–6 copies of *pfmdr*1 gene. Although increase in *pfmdr*1 gene expression has been reported in Africa, the frequency and the copy number of *pfmdr*1 gene in African parasite population is usually low compared to that present in SEA parasites^10^,^21^,^22^,^25^,^26^,^27^,^28^. Further, increase in *pfmdr*1 gene expression has been shown to be present in African parasites but with inconsistent frequency longitudinally^22^,^27^. In a recent efficacy study conducted in Sudan, amplification of *pfmdr*1 gene was shown to contribute to recurrent *P. falciparum* parasitemia following AL treatment^21^. Data from our study showed *pfmdr*1 gene copy numbers increased longitudinally in samples collected between pre- and post-ACTs periods, consistent with previous studies^21^,^22^. Further, we observed significant association of increased *pfmdr*1 gene expression with reduced in vitro drug susceptibility to amodiaquine and piperaquine. Based on molecular markers, studies have shown chloroquine resistance remains high in western Kenya^29^,^30^. Amodiaquine, chloroquine and piperaquine are in the 4-aminoquinoline drug family, which might explain the significant association seen of increased *pfmdr*1 gene expression with reduced in vitro susceptibility to amodiaquine and piperaquine. This contradicts a study conducted in Kenya where *pfmdr*1 copy number was not associated with amodiaquine resistance^26^. This can be explained by the fact that our data is from an in vitro study whereas the previous report was from an in vivo study. Additional in vitro and in vivo studies are required to further clarify data observed thus far in Kenyan samples.

We did not observe any association between *pfmdr*1 gene expression with in vitro drug activity to lumefantrine, mefloquine or the artemisinin derivatives. Increase in *pfmdr*1 copy number has been shown to be the most relevant determinant of in vitro and in vivo resistance to mefloquine and lumefantrine in SEA both which are aryl amino alcohols^10^,^11^. However, *pfmdr*1 amplification is rarely reported in African parasites^25^,^26^,^27^, with only one clinical case which showed a direct link between *pfmdr*1 gene copy number and mefloquine clinical resistance^31^. A recent efficacy study showed amplification of *pfmdr*1 gene contributes to recurrent *P. falciparum* parasitemia following AL treatment^21^. In our analysis, we could not ascertain this observation because none of the recurrent parasites had more than one copy of the *pfmdr*1. The difference seen between *pfmdr*1 resistance phenotypes of SEA and African parasites can be explained by difference in parasite population genetic background and profiles.

In conclusion, we have studied SNPs described by Cheeseman *et al*^8^ in *P. falciparum* parasite population from Kenya. Data indicates these SNPs were present before the introduction of ACTs but declined with introduction of ACTs, which implies the pressure that caused existence of these SNPs was removed in post-ACTs period, causing the parasite to revert back to the wild type. The pressure is likely to have come from chloroquine or SP, which were the first-line antimalarial drugs when the pre-ACTs samples were collected. Further, we have shown increased amplification of *pfmdr*1 gene copy number overtime, with statistical significance between pre- and post-ACTs sample isolates. Majority of the samples with multiple *pfmdr*1 copy number were post-ACTs. However, there was no association of increased *pfmdr*1 copy number with recurrence of *P. falciparum* parasitemia following AL treatment.

## Methods

### Samples

The pre-ACTs isolates used in this study were from cryopreserved archived samples collected in Kisumu County, western Kenya between 1995 and 2003. This was before AL was adopted as the first-line treatment for uncomplicated malaria in Kenya. The post-ACTs samples were collected from a randomized, two-arm open-label trial (ClinicalTrials.gov Identifier: NCT01976780) conducted in 2013–2014 in Kombewa district in Kisumu County, western Kenya; eight years after AL was rolled-out as the first-line treatment for uncomplicated malaria. Both studies were carried out in accordance to approved guidelines by the Ethical Review Committee of the Kenya Medical Research Institute (KEMRI), Nairobi, Kenya and Walter Reed Army Institute of Research (WRAIR) Institutional Review Board, Silver Spring, MD. The pre-ACTs study and post-ACTs studies were conducted under approved study protocols KEMRI-SSC 1330/WRAIR 1384 and KEMRI-SSC 2518/WRAIR 1935 respectively. In both studies, patients presenting with uncomplicated malaria, aged between 6 months and 65 years were consented and recruited into the studies; written informed consent was provided by study participants and/or their legal guardians. The presence of malaria was confirmed by microscopy. Whole blood was collected and aliquots preserved for analysis as specified in the study protocols.

### Parasite genotyping

Genomic DNA was extracted from whole blood using QIAamp Blood mini kit (Qiagen, Valencia, CA) as recommended by the manufacturer. Extracted DNA was stored appropriately until analyzed. Twenty five single nucleotide polymorphisms (SNPs) in chromosome 13 and 14 of *P. falciparum* genome previously described by Cheeseman *et al*^8^ were analyzed using PCR-based single-base extension on Sequenom MassARRAY system (Sequenom, San Diego, CA) following manufacturer recommendations. The primers used in the analysis of the 25 SNPs can be found in the Supplementary Table S1. Additional two SNPs were analyzed using PCR-Restriction Length Fragment Polymorphism (RLFP) as described by Takala-Harrison *et al*^18^, following modified PCR_RLFP protocol as described in worldwide antimalarial resistance network (WWARN) website. The SNPs analyzed using this technique were MAL10-688956 and MAL13-1718319 v1.0.

### Estimation of *pfmdr*1 copy number

The *pfmdr*1 copy numbers were estimated as previously described^10^ using ABI 7500 real-time fast qPCR system (Applied Biosystems Inc., Foster City, CA). For each isolate, two-three independent experiments were ran, with each run performed in triplicate and analyzed with SDS software (version 2.0.6; Applied Biosystems Inc., Foster City, CA). Reference strains Dd2 (which carries 2–3 *pfmdr*1 gene copies) and 3D7 (which carries one *pfmdr*1 gene copy) were used as controls in each run. The 2^−ΔΔCT^ relative quantification technique was used to estimate copy number variation. Isolates with copy number ≥ 1.75 were considered to have true *pfmdr*1 gene amplification.

### In vivo clearance of *Plasmodium* falciparum

Parasitemia was estimated every 6 hours by examining Giemsa-stained thin and thick blood smears in 200 high power fields. For quantification, parasite density per micro liter was estimated from the number of parasites counted per 2000 WBCs and based on the total number of WBCs from the complete blood count. Parasite half-lives were calculated as log (2)/CR as previously described^32^.

### In vitro drug sensitivity tests

Drugs used for in vitro testing including artemether (AT), artesunate (AS), dihydroartemisinin (DHA), amodiaquine (AQ), lumefantrine (LU), mefloquine (MQ) and piperaquine (PPQ) were obtained from WRAIR. Parasites were maintained in continuous culture as previously described^33^ and tested for in vitro susceptibility by SYBR Green I- based assay^30^,^34^,^35^,^36^. *Plasmodium falciparum* parasites in continuous culture attaining 3 to 8% parasitemia were adjusted to 2% hematocrit and 1% parasitemia. The field isolates were assayed by immediate *ex vivo* as previously described^30^,^36^. Drugs were dissolved in dimethyl-sulfoxide to attain 5 mg/mL. These were diluted to working concentrations that then underwent two-fold serial dilutions across 10-concentrations ranging from (in nM) AT 335 to 0.65, AS 260.2 to 0.51, DHA 352 to 0.69, AQ 230 to 0.45, LU 189 to 0.37, MQ 602.7 to 1.18 and PPQ 270 to 0.52 on 96-well microliter plates as previously described^30^. The assay was initiated by addition of 100 μL reconstituted parasite components to each well of the drug plates and incubation at 37°C as previously described^30^,^34^,^36^. The assay was terminated after 72 hours by adding 100 μL of lysis buffer containing SYBR green I (20x final concentration) directly to the plates and kept at room temperature in the dark, for 24 hours. The 50% inhibition concentration (IC_50_) values for each drug was calculated as described previously^30^,^36^.

### Data analysis

MassARRAY Workstation software was used to process and analyze iPLEX Spectro CHIP bioarrays. Spectro TYPER-RT (Sequenom) software was used to calculate the expected position of the correct analyte peaks in the spectra Call. SNPs with minor allele frequency (MAF) of < 1% were not considered for further analysis; those with MAF > 5% and < 10% missing data were used for analysis. Continuous data generated were expressed as medians with Interquartile Ranges (IQR) or, in the case of parasite counts, as geometric means with 95% confidence intervals (95% CI). Differences in clearance rates or in vitro susceptibility based on suspected genotype profiles were compared using the non-parametric 1-way ANOVA, Kruskal-Wallis with Dunn's multiple comparison post test and Mann-Whitney test as appropriate. Significance was defined as p value (p < 0.05). Statistical analyses were performed using Graphpad Prism, version 5.0.2; (Graphpad Software, Inc., San Diego, CA) and Stata version 11 (College Station, TX, USA).

## Author Contributions

E.K., conceived, designed, supervised the project; wrote the manuscript. H.M.A. supervised in vitro experiments, assisted in data analysis and manuscript review. B.A., was the principle investigator for the efficacy study; provided samples and advised on data analysis. Z.N. supervised project activities and provided guidance on the analysis plan. A.O.A. supervised laboratory experiments. D.W.J., P.M., R.A.Y., J.C., E.M., G.M., A.C.C. and L.J.C., assisted in performing experiments. I.O. and J.O., recruited subjects, provided the field samples and clearance data, L.A.I. and B.S.N., conceived, contributed study design, performed experiments, analyzed the data and manuscript writing.

## Supplementary Material

Supplementary Information Supplementary Information

## Figures and Tables

**Table 1:** Single nucleotide polymorphism profile and respective minor allele frequencies in different years

Combined SNP-ID	Kisumu samples 1995–2003	Kisumu samples 2005–2010	Kisumu clinical trial samples 2013–2014
	n = 48 Allele (MAF)	n = 10 Allele (MAF)	n = 71 Allele (MAF)
**MAL13_4043**	C (0.06)		
**MAL13_5817**	T (0.06)		T(0.01)
**MAL13_5862**	G (0.08)		
**MAL13_5938**	G (0.02)		
**MAL13_6069**	C (0.06)		
**MAL13_6117**	G (0.10)		
**MAL13_6300**	C (0.25)	C(0.1)	C(0.13)
**MAL14_8449**	A (0.04)		A(0.03)
**MAL14_8696**	C (0.06)		
**MAL14_8697**	C (0.02)		
**MAL14_8702**	C (0.44)	C(0.4)	C(0.31)
**MAL14_8744**	G (0.08)		
**MAL14_9356**	G (0.06)		
**MAL14_9360**	T (0.54)	T(0.3)	T(0.38)
**MAL14_9361**	T (0.13)	T(0.4)	T(0.01)

**Table 2:** Correlation between IC_50_ and *pfmdr*1copy number outcome

	Single *pfmdr*1 copy	Multiple *pfmdr*1 copy	
Drug	Median IC_50_ (IQR) nM	Median IC_50_ (IQR) nM	p value
**Artemether**	3.77 (2.7–5.49)	4.21 (3.21–5.8)	0.36
**Artesunate**	15.01 (4.39–22.15)	11.51 (9.93–16.53)	0.80
**Dihydroartemisinin**	5.83 (0.0–12.09)	7.071(5.62–11.35)	0.59
**Amodiaquine**	3.93 (0.33–5.67)	8.62 (6.76–18.17)	0.009
**Lumefantrine**	29.9 (0.74–43.12)	56.12 (28.11–48.35)	0.26
**Mefloquine**	2.14 (0.98–3.14)	4.02 (1.79 –8.22)	0.21
**Piperaquine**	26.37 (0.72–35.46)	37.29 (24.97–177.8)	0.038
