# Supplementary material for: Analysis of Major Genome Loci Underlying Artemisinin Resistance and pfmdr1 Copy Number in pre- and post-ACTs in Western Kenya
Source: Sci Rep. 2015 Feb 6;5:8308. doi: 10.1038/srep08308 (PMC4319159; doi:10.1038/srep08308)
Supplement: Supplementary Information [file srep08308-s1.pdf]

Analysis of Major Genome Loci Underlying Artemisinin Resistance and *pfmdr*1 Copy Number in pre- and post-ACTs in Western Kenya

Bidii S. Ngalah[1, 2], Luiser A. Ingasia[1], Agnes C. Cheruiyot[1], Lorna J. Chebon[1, 2], Dennis W. Juma[1], Peninah Muiruri[1,2], Irene Onyango[1], Jack Ogony[1], Redemptah A. Yeda[1], Jelagat Cheruiyot[1,3], Emmanuel Mbuba[4], Grace Mwangoka[4], Angela O. Achieng[1,3], Zipporah Ng'ang'a[2], Ben Andagalu[1], Hoseah M. Akala[1], Edwin Kamau[1*]

Supplementary Information:

Supplementary note: Additional information on in vivo efficacy study where clearance half-life of the parasite infection to artemether-lumefantrine was obtained.

Supplementary Table S1: Primers used in analysis of the 25 SNPs on Sequenom MassARRAY system. The primers were generated by the design software which is part of the Sequenom MassARRAY system. The 25 SNPs were designed into 2 pools.

Supplementary Note:

Study Design

This was a two-arm randomized open-label trial. The protocol was approved by KEMRI and WRAIR Institutional Review Boards. The approved protocols are KEMRI, SSC protocol No 2518 and WRAIR # 1935. The study was conducted in accordance with the Declaration of Helsinki (2002), Good Clinical Practices guidelines set up by the International Conference on Harmonization[1], and local applicable laws and regulations. Eligible study subjects were open guided through the informed consent form after which those who consented were enrolled into the study.

The study aimed to assess the degree of artemisinin resistance in subjects presenting with uncomplicated *P. falciparum* malaria in western Kenya. 118 individuals aged between 6 months and 65 years inclusive (minimum weight 11kg), presenting with a measured temperature of ≥ 37.5^0C or history of fever within 24 hours prior to presentation and with mono-infection with *Plasmodium falciparum* (baseline parasitemia of 2000-200,000 asexual parasites/µl) were enrolled. Subjects were excluded if they had severe and/or complicated malaria, including severe anaemia (haemoglobin (Hb) ≤5 g/dL). Subjects were then randomized to receive either artesunate-mefloquine or artemether lumefantrine at the standard dosage. Data from the artemether lumefantrine arm was used for the current analysis.

Assessment

Venous blood samples for malaria blood films were collected at hours 0, 4, 8, 12, 18, 24 and then 6 hourly until 2 consecutive negative smears were obtained. Two independent expert microscopists examined the Giemsa-stained films and any discrepancies resolved by involving a

third expert reader then taking the findings of the 2 closest readers. At least 200 high power fields were examined before a smear was considered negative. Parasitemia per microliter of blood was calculated using the actual white cell or red cell count (for low and high parasitemia respectively) obtained from a complete blood count run from a sample drawn on the same day as the malaria film. The geometric mean of the parasite count per microliter from the 2 readers for each subject at each sampling time point was then calculated. Patients were followed for a total of 42 days with contacts as follows: day 7, 14, 21, 28, 35, and 42. During the follow-up visits, physical examination was performed and vital signs including axillary temperature recorded. Blood was collected for complete blood count (CBC) assay, genotyping and malaria diagnosis by malaria blood smear reading. In case of parasite reappearance, malarone was administered as the rescue treatment. Adverse events (AEs) and serious adverse events (SAEs) were recorded and monitored throughout the study.

Data analysis

PCR analysis on merozoite surface protein (msp)-1, and msp-2 was done to distinguish recrudescent parasites from new infections by capillary electrophoresis (CE). Genotyping was done as previously published[2] by CE on an ABI 3500xL genetic analyzer (Applied Biosystems, Foster city, CA) as described in the manufacturer user guide. Parasite clearance rates were calculated using WorldWide Antimalarial Resistance Network (WWARN) tool for Parasite Clearance Estimator (PCE) on their website (http://www.wwarn.org/toolkit/data-management/parasite-clearance-estimator). Log transformed parasite density was plotted against time in hours to generate slope half-life, the time needed for parasitemia to be reduced by half. This constant is independent of starting value of parasitemia. The half-life was calculated as follows:

$T_{1/2} = \log_e (2)/k$

$= 0.692/k$, where k is the clearance rate constant

References

1. ICH Harmonized Tripartite guideline: Guidelines for Good Clinical practiceE6 (R1);1996. Current Step 4 version. http://www.ich.org/fileadmin/Public_Web_Site/ICH_Products/Guidelines/Efficacy/E6_R1/Step4/E6_R1__Guideline.pdf.
2. Liljander et al. Optimization and validation of multi-colored capillary electrophoresis for genotyping of Plasmodium falciparum merozoite surface proteins (msp1 and 2) Malar J. 2009;8:78

Supplementary Table S1

Primers used for analysis of 25 SNPs as described by Cheeseman *et al*[1].

SNP_ID	1st-PCRP	2nd-PCRP	UEP_SEQ
CombinedSNP_MAL13_1823	ACGTTGGATGGCAATATTCAATGTGAGGAAG	ACGTTGGATGCTTTAAAGAAAAACTACTAG	cTAAAGAAAAACTACTAGTACCAA
CombinedSNP_MAL13_2384	ACGTTGGATGTGGTAATAATATTATTGATG	ACGTTGGATGAATTTGAGGATATGATTATC	GAGGGACAAATTTTCCATT
CombinedSNP_MAL13_3474	ACGTTGGATGAGAGCATGAGAATAATGTGG	ACGTTGGATGCCATTTTCATCGTATGTTCC	TATTGTATGTTCCATTTTTATCATAT
CombinedSNP_MAL13_4041	ACGTTGGATGTTCAAAAGTTGCAGGTGAAG	ACGTTGGATGACTTTTTTTCGACATCTAC	cTTCGACATCTACTTTTTTTATACTGA
CombinedSNP_MAL13_4043	ACGTTGGATGGATCATCTTTTAAATTTTCAAC	ACGTTGGATGCTTTTCAAGTATTTGTAATG	TTTTCAAGTATTTGTAATGATTTAAC
CombinedSNP_MAL13_5817	ACGTTGGATGGGTTTATCTGTATTCAAATT	ACGTTGGATGCAAGTAAAGTTCTAGATGAAG	gAAAGTTCTAGATGAAGAAAAATTAA
CombinedSNP_MAL13_5862	ACGTTGGATGTGACGGTGCATATTTTTTG	ACGTTGGATGCAAAGCTGTCTCTTTTCTTTC	ccTGAAATCCTTCACCAAAATA
CombinedSNP_MAL13_5938	ACGTTGGATGATTTTTTATGATCATTAAAC	ACGTTGGATGAATGAATTGGAAACTTACC	ATTGGAAACTTACCATAGTTTGC
CombinedSNP_MAL13_6016	ACGTTGGATGCGTAAACATATACTCTAATG	ACGTTGGATGTTAAATTATGCTCACATGG	aACATGGAAAATTTTAAAAGTAATAATA
CombinedSNP_MAL13_6069	ACGTTGGATGATGAATAAATAACATAATGC	ACGTTGGATGGTTATGGCAAATGCAACAGG	ATGCAACAGGTCTATATATTCA
CombinedSNP_MAL13_6116	ACGTTGGATGTGTATATCTTGCTTTTTTAC	ACGTTGGATGTCCTAATTCGAACGAAAAC	TTCGAACGAAAACATATCAAC
CombinedSNP_MAL13_6117	ACGTTGGATGCTTGTTTCTTTTGTTCTTC	ACGTTGGATGGTTTGTTTGTTTGTTTGTTTG	TGTTTATTTGATTTAACAAAATGATG
CombinedSNP_MAL13_6300	ACGTTGGATGGTAATAAATTGAATTTTCCC	ACGTTGGATGGGAGATGTATCATTTGCACG	TGCACGAACTCCTTG
CombinedSNP_MAL13_6305	ACGTTGGATGCCATTTCCATATATTTCTTC	ACGTTGGATGGAATAGTTGTGACAAATCTG	ccgacTAGTTGTGACAAATCTGTTACAT
CombinedSNP_MAL14_8445	ACGTTGGATGGGATATTAAAATTCATGTAG	ACGTTGGATGGCTCATCTGGTTTATTAGTTG	ATAAATTATCTGTGTCACAAAATTC
CombinedSNP_MAL14_8449	ACGTTGGATGTTTTTTTTCAAAGGATTTTC	ACGTTGGATGGAGCTCAAAGGTATAATAGG	aTCAAAGGTATAATAGGAAATTCA
CombinedSNP_MAL14_8696	ACGTTGGATGTTTTCTATATTACTAATAGC	ACGTTGGATGCTGCTGACTTTTTGTGGATG	ACTTTTTGTGGATGATTATTTTAT
CombinedSNP_MAL14_8697	ACGTTGGATGACTAATAGCATACAACAAG	ACGTTGGATGTTGTAGCTCGTTAGAAGAGG	ACAATTCTGCTGACTTTTT
CombinedSNP_MAL14_8702	ACGTTGGATGGGATTACTTTCATATGTATT	ACGTTGGATGTTTTGATTTTGAAAATGCTC	aTGATTTTGAAAATGCTCTATATATAAG
CombinedSNP_MAL14_8744	ACGTTGGATGCACCAAGTCAAATTACAACC	ACGTTGGATGTGCCTAGTATGTCCTACAAC	ggTTGCATTATATTTTTTAAGAATGTTT
CombinedSNP_MAL14_8769	ACGTTGGATGATTCCTTATTATCCATGAC	ACGTTGGATGCACAAATAGTTATAATAGTGG	ccccATAATAGTGGATCCTCTACAC
CombinedSNP_MAL14_9356	ACGTTGGATGTCCGATTGAAAGACGCAAAC	ACGTTGGATGCAAAGAGTAGAAATATATGG	TATGGATTTTCTATTTTTTTTAATTCTG
CombinedSNP_MAL14_9357	ACGTTGGATGAGTTATAGTAGTTTAATCTG	ACGTTGGATGATTTGTGTAACCACACAAGC	CACACAAGCATTCGATT
CombinedSNP_MAL14_9360	ACGTTGGATGGCTTCATGATATGAACCGAG	ACGTTGGATGCATCTTGAATAAGTCATCCG	CCGTTAAAACATTCTGAAGAG
CombinedSNP_MAL14_9361	ACGTTGGATGTGAAGCGGGGCACTATTTAC	ACGTTGGATGGAGTGATATCCTAGAGCACA	tcgcGCACATAAGATTGTTCTTTTTTT

1. Cheeseman, I. H. et al. A major genome region underlying artemisinin resistance in malaria. Science 336, 79-82 (2012).
